# A rapid malaria appraisal in the Venezuelan Amazon

**DOI:** 10.1186/1475-2875-8-291

**Published:** 2009-12-11

**Authors:** Wolfram G Metzger, Anibal M Giron, Sarai Vivas-Martínez, Julio González, Antonio J Charrasco, Benjamin G Mordmüller, Magda Magris

**Affiliations:** 1 Servicio Autónomo Centro Amazónico para la Investigación y Control de Enfermedades Tropicales, Simón Bolívar' (SACAICET), Puerto Ayacucho, Estado Amazonas, Venezuela; 2 Dirección Regional de Salud Amazonas, Ministerio del Poder Popular para la Salud, Puerto Ayacucho, Estado Amazonas, Venezuela; 3 Cátedra de Salud Pública. Escuela de Medicina Luis Razetti. Universidad Central de Venezuela, Caracas, Venezuela; 4 Eberhard Karls Universität Tübingen, Institut für Tropenmedizin, Tübingen, Germany

## Abstract

**Background:**

While the federal state of Amazonas bears the highest risk for malaria in Venezuela (2007: 68.4 cases/1000 inhabitants), little comprehensive information about the malaria situation is available from this area. The purpose of this rapid malaria appraisal (RMA) was to provide baseline data about malaria and malaria control in Amazonas.

**Methods:**

The RMA methodology corresponds to a rapid health impact assessment (HIA) as described in the 1999 Gothenburg consensus. In conjunction with the actors of the malaria surveillance system, all useful data and information, which were accessible within a limited time-frame of five visits to Amazonas, were collected, analysed and interpreted.

**Results:**

Mortality from malaria is low (< 1 in 10^5^) and slide positivity rates have stayed at the same level for the last two decades (15% ± 6% (SD)). Active case detection accounts for ca. 40% of slides taken. The coverage of the censured population with malaria notification points (NPs) has been achieved in recent years. The main parasite is *Plasmodium vivax *(84% of cases). The proportion of *Plasmodium falciparum *is on the decline, possibly driven by the introduction of cost-free artemisinin-based combination therapy (ACT) (1988: 33.4%; 2007: 15.4%). Monitoring and documentation is complete, systematic and consistent, but poorly digitalized. Malaria transmission displayed a visible lag behind rainfall in the capital municipality of Atures, but not in the other municipalities. In comparison to reference microscopy, quality of field microscopy and rapid diagnostic tests (RDTs) is suboptimal (kappa < 0.75). Hot spots of malaria risk were seen in some indigenous ethnic groups. Conflicting strategies in respect of training of community health workers (CHW) and the introduction of new diagnostic tools (RDTs) were observed.

**Conclusion:**

Malaria control is possible, even in tropical rain forest areas, if the health system is working adequately. Interventions have to be carefully designed and the features of the particular local Latin American context considered.

## Background

Routine malaria surveillance systems, and the data they produce, need to be analysed, otherwise many useful data may become lost. Yet it is important to note that, outside of experimental trials, the effectiveness of interventions such as, for example, insecticide-treated bed nets, can only be evaluated when routine baseline data has already been assessed. In 2007, a rapid malaria appraisal (RMA) was carried out as part of a project providing technical assistance to the malaria surveillance system in the Federal State of Amazonas, Venezuela. The purpose of this project was to provide baseline data and facilitate operational changes. In what follows, results of this evaluation are presented in brief.

The Federal State of Amazonas (180,145 km^2^) covers an area as big as the UK, excluding Scotland. More than half of the 135,585 inhabitants live in the capital Puerto Ayacucho, which is situated in the far North-West of the territory. Amazonas is the most southern federal state of Venezuela, bordering Colombia to the west and Brazil to the east. It comprises seven municipalities characterized by very low density of population, remoteness of communities, absence of roads, and transport via river or air. Half of the population originates from 19 indigenous ethnic groups. In the municipality of *Alto Orinoco *live about 12,000 Yanomami people, 5,000 of whom it is estimated continue to reside in the vast expanse of the tropical forest remaining uncaptured by any census [1]. Unlike the neighbouring state of Bolivar, Amazonas does not host significant gold mining activities.

The malaria surveillance system in Venezuela was founded in 1936 by Arnoldo Gabaldón, who became a legendary figure in the history of public health in Venezuela. For a long time this institution (known as *malariologia*) acted with a high degree of autonomy. Based on DDT spraying, early diagnosis and therapy, it was designed as a vertical programme with teams of health workers visiting the communities. The drug distribution service was probably the first of its type to make anti-malarials available, without payment, to rich and poor alike [2]. Partly as result of these activities, in 1961 WHO declared the eradication of malaria in the major part (2/3) of the then malarious areas of Venezuela [3]. However, the Amazon region was excluded from eradication efforts because malaria there was designated as inaccessible.

Malaria research activities in Amazonas have been infrequent, but have increased in recent years. A search for peer-reviewed articles containing data on clinical malaria, mainly from cross-sectional studies, listed 18 publications from 1990 onward [4-21]. The majority (12/18) were carried out in the Yanomami people of the southern municipality *Alto Orinoco*. Very few data were from Atures, even though most malaria cases are from this northern municipality (see also Malaria Atlas Project 2007 [22]). Entomological data are presented in various publications describing *Anopheles darlingi *as the primary vector [17].

In 2002, the malaria surveillance system was integrated by the Ministry of Health into the main epidemiological surveillance system. As for supporting infrastructure, to date, the governmental health sector of Amazonas counts one hospital constructed in the 1950s (100 beds), one modern Popular Clinic, six Comprehensive Health Centres in construction, six Rehabilitation Centers, seven urban and 92 rural health posts, as well as 32 health posts of the *Barrio Adentro *programme [23,24]. A modern hospital (220 beds) is under construction and will be completed in 2010. The Venezuelan constitution (1999) guarantees cost-free medical care and forbids the privatization of the public health system.

## Methods

This work was done within the framework of providing technical assistance to the malaria surveillance system of the federal state of Amazonas, Venezuela, carried out between October 2007 and August 2008. The study represented a rapid malaria appraisal (RMA) corresponding to a rapid health impact appraisal (HIA) under resource-constrained conditions [25]. Established guidelines for monitoring and evaluation [26] were followed and modified according to local needs. The study comprised retrospective, concurrent and prospective aspects.

Five visits were carried out. The duration of each was between five days and five weeks. Field investigations were undertaken in the capital municipality of *Atures*, and short visits were carried out to the municipalities of *Autana*, *Atabapo*, *Manapiare*, and *Alto Orinoco*. The documentation system was analysed, data were extracted and additional data were collected. Structured and semi-structured questionnaires were applied with individual and group interviews being conducted. A pilot study to assess the quality of malaria diagnoses (microscopy, Rapid Diagnostic Tests) was carried out and workshops were held at the end of each visit. All statistical analyses were done using R v2.7 [27]. Time series were analysed for trend and serial dependence.

## Results

### Malaria in Venezuela and Amazonas, 1988 - 2007

**Figure 1:**
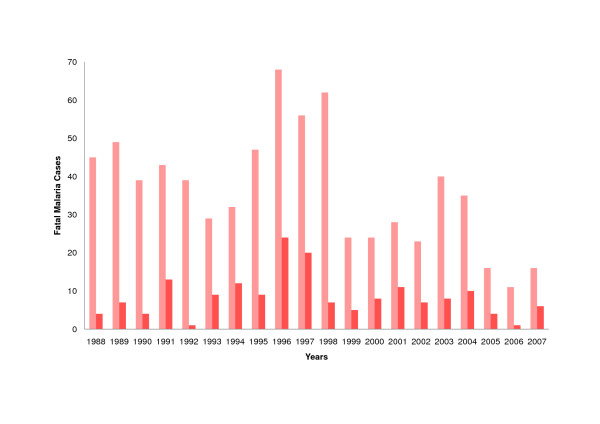
**Malaria Mortality in Venezuela and Amazonas (1988-2007)**. Fatal malaria cases in Venezuela (bright red), fatal malaria cases in Amazonas (dark red).

In Venezuela, the number of malaria cases increased within two decades from 22,056 in 1988 to 41,570 in 2007 displaying a peak in 2004 (46,244 cases). Whilst roughly 20% of malaria cases were due to *Plasmodium falciparum*, overwhelmingly the rest (nearly 80%) were attributed to *Plasmodium vivax*. Interestingly, *Plasmodium malariae *and mixed infections are only marginally detected. Mortality from malaria (using ICD-9 (084) and ICD-10 (B50-B54) is low and fell significantly from 45 (1988) to 16 (2007) fatal cases. A linear model of the data showed a decrease of 1.47 deaths per year (Figure 1) (p = 0.01) [28]. In 2007, Venezuela counted with a population of 26,02 × 10^6^million inhabitants [29] resulting in a countrywide mortality rate from malaria of < 1 in 10^6 ^inhabitants.

Amazonas contributes about a fifth (2007: 22%) of the total of malaria cases in Venezuela and ranks second, in regard to absolute case numbers, behind Bolivar. However, due to its low population density, it displays the highest incidence per head of population (2007: 68.4 cases/1,000 inhabitants). The analysis of mortality records shows that mortality due to malaria in Amazonas is low and has remained at the same level for the last twenty years (p = 0.68) (Figure 1) [28].

**Figure 2:**
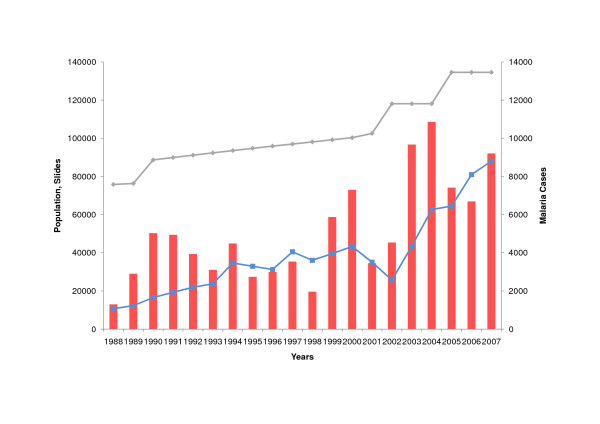
**Malaria Cases, Slides taken, Population in Amazonas (1988-2007)**. Malaria cases (red, right vertical axis), slides taken (blue, left vertical axist), population (grey, left vertical axis).

In the same period, the number of malaria cases detected in Amazonas multiplied nearly nine times (1988: 1,297 cases; 2007: 9,204 cases). Within this period, the population almost doubled (1988: 75,830 inhabitants; 2007: 134,585 inhabitants) and the number of slides taken increased more than eight times (1988: 10,736 slides; 2007: 88,079). Also the coverage of permanent active malaria notification points (NPs) rose from 2 NPs (1988) to 82 NPs (2007). The majority of NPs were established between 2003 and 2005, which is reflected in the increase in slides taken within that period (Figure 2).

**Figure 3:**
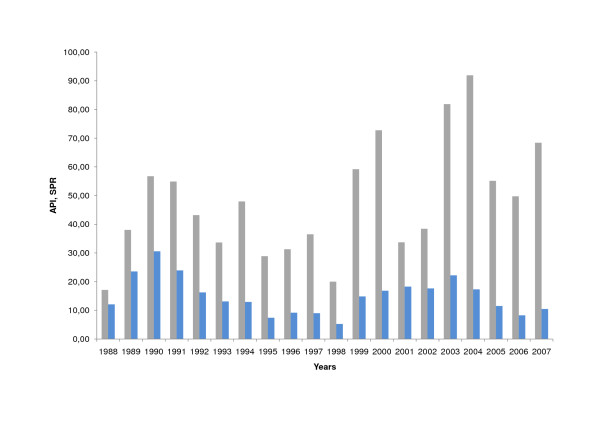
**Annual Parasite Index and Slide Positivity Rate in Amazonas (1988 - 2007)**. Annual parasite index (grey, API), slide positivity rate (blue, SPR).

Consequently, the annual parasite index (API, malaria cases per 1,000 persons per year) for malaria cases increased from 17 (1988) to 68 (2007) (p = 0.021), but the slide positivity rate (SPR, malaria cases per 100 slides taken) did not change significantly for the last 20 years (SPR 1988: 12; SPR 2007: 10) (p = 0.17) (Figure 3).

**Figure 4:**
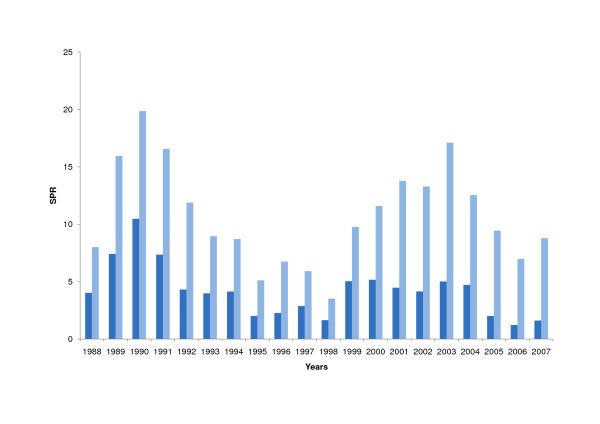
**Slide Positivity Rate of *P. falciparum *and *P. vivax *in Amazonas (1988 - 2007)**. Slide positivity rate (SPR) of *P. vivax *(bright blue), slide positivity rate (SPR) of *P. falciparum *(dark blue).

Subgrouping for malaria species revealed that the percentage of *P. falciparum *decreased steadily (1988: 33.4%; 2007: 15.4%) which is reflected in different patterns of the SPR for the two species. The SPR of *P. vivax *remained on the same level (p = 0.43), whereas the SPR of *P. falciparum *decreased significantly (p = 0.018). (Figure 4).

### Rainfall and malaria transmission in Amazonas municipalities, 1998 - 2007

**Figure 5:**
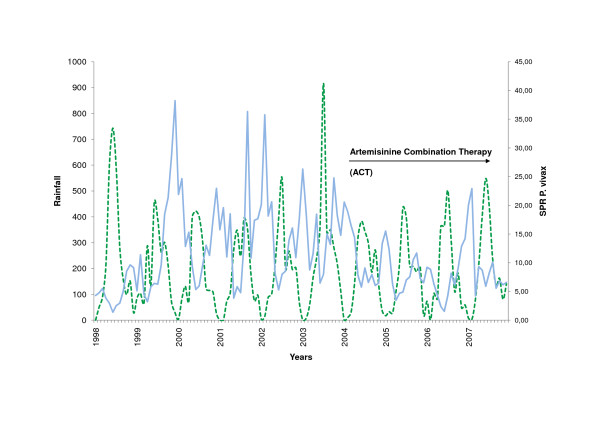
***P. vivax *Slide Positivity Rate and Rainfall in Atures/Amazonas (1998 - 2007)**. Slide positivity rate *P. vivax *(blue, SPR), rainfall (green).

**Figure 6:**
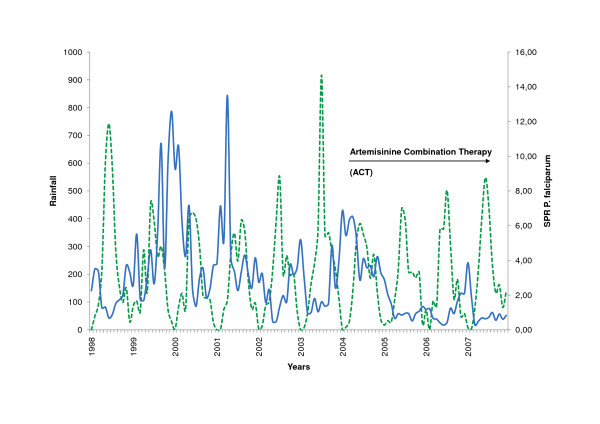
***P. falc*. Slide Positivity Rate and Rainfall in Atures/Amazonas (1998 - 2007)**. Slide positivity rate *P. falciparum *(blue, SPR), rainfall (green).

Subanalysis for the municipalities of Amazonas could be carried out for the decade 1998 to 2007 only, because prior to this period a reform at local government level had been undertaken, changing names and territory of some of the municipalities. Malaria transmission displayed a visible lag behind peaks of rainfall in the capital municipality of Atures (Figure 5, 6), which was not seen in the other municipalities. Partial autocorrelation of datasets showed a significant positive correlation of rainfall with the incidence of *P. vivax *with a lag of 4 months. This pattern was even more pronounced in *P. falciparum *where parasite incidence was significantly lagging two to five months behind rainfall. Negative correlations of *P. falciparum*, that fit the expected seasonal pattern, could be seen 1, 7, 10, and 11 months after rainfall.

### Malaria species, sex, age and ethnic differences, 2007

In 2007, out of the 9,204 cases found positive for malaria in Amazonas, 447 (4.8%) were imported, mainly from Colombia. Out of the autochthonous cases (n = 8757), more than half of the patients came from the municipality of Atures, the rest were distributed among the other municipalities (Table 1) [30].

**Table 1:** Malaria and malaria control in Amazonas

	Population	Malaria Cases	NP*	Microscopists	Visitors	Inspectors
Atures	86,368	4,342	32	13	10	4

Autana	7,693	1,339	12	7	3	1

Atabapo	9,991	1,005	6	3	2	1

Manapiare	9,063	813	19	10	2	1

Alto Orinoco	17,058	1,165	11	3	5	1

Maroa	1,848	5	1	1	0	0

Rio Negro	2,564	88	1	1	0	0

* Notification Point

**Figure 7:**
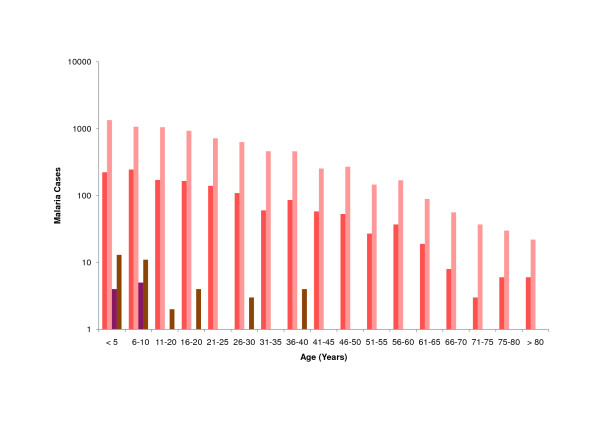
**Malaria Infections by Age Groups in Amazonas (2007)**. Malaria cases of *P. falciparum *(dark red), *P. vivax *(bright red), *P. malariae *(purple), mixed infections (brown).

7736 cases (83%) were caused by *P. vivax*, 1416 (16%) cases were caused by *P. falciparum*, 12 cases (0.1%) cases were attributed to *P. malariae*, and 40 cases (0.4%) were mixed infections. Interestingly, the distribution of species showed significant differences between ethnic groups. For example, the Yanomami group displayed a significantly higher rate of *P. falciparum *infections than the other ethnic groups (Yanomami 40.3%, Yekwana 22.4%, Puinabe 16.2%, Guahibo 14.9%, Baré 12.1%, Criollo 11.2%, Curripaco 10.9%, Piapaco 9.2%, Piaroa 8.7%). Subgrouping by age groups showed that 17,2% of cases were in patients under five years of age (Figure 7).

**Figure 8:**
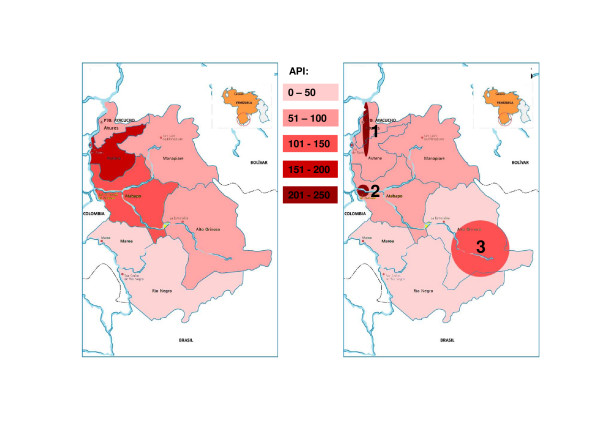
**Annual Parasite Index (API) by Municipalities and by Ethnic Groups in Amazonas (2007)**. Left: Annual Parasite Index (API) by municipalities. Right: Annual Parasite Index (API) by ethnic groups. Schematic illustration of the settlement area of the ethnic groups of Guahibo (1), Puinave (2), Yanomami (3).

The overall risk of malaria showed considerable differences between the municipalities. This picture changed noticeably when ethnic groups were mapped and a higher risk was revealed for some ethnic groups in comparison to the neighbouring population (Figure 8).

### Structure of the malaria surveillance system in Amazonas

The malaria surveillance system in Amazonas employs around 150 persons, half of them with renewable short-term contracts. It is embedded in the Service of Environmental Surveillance which runs also the control of dengue and intestinal parasites. Malaria Control is headed by a coordinator and divided into sections of Vector Control, Medical Attention and Entomology. The main actors are inspectors (two years training in the central school for malaria inspectors in Maracay, Venezuela, microscopists (three to six months training as auxiliary nurses), visitors and fumigators (two months training as auxiliary staff). The distribution of malaria personnel reflects roughly the population density of the municipalities (Table 1) [30].

Aside from the traditional system, the 'Andean Plan for Malaria in Frontier Areas' (PAMAFRO, *Plan Andino de Malaria en Areas Fronterizas*) has been operating in Venezuela since 2005. This non-governmental organization is financed by the Global Fund for the Fight against AIDS, Tuberculosis and Malaria. One focus of its strategy is the participation of the communities on a voluntary basis [31]. To date, about 150 community health workers have been trained within this programme.

The states surveillance system and PAMAFRO, operated quasi-independently of each other until 2008, when PAMAFRO was integrated into the central coordination of the malaria surveillance system.

### Knowledge of malaria staff about malaria

An anonymous self-made questionnaire was given out to explore the knowledge about malaria of the personnel (technical and non-technical) working in the control system of the municipality of Atures. Answers to six multiple choice questions about malaria were requested (see additional file 1). Out of 75 employees, 52 returned their sheets fully completed. Results were, in brief: 1) 92% (48/52) knew the transmission cycle of Plasmodium. 2) 54% (28/52) could identify the three types of malaria in Venezuela. 3) 73% (38/52) identified *P. falciparum *as the most dangerous of the parasites. 4) 46% (42/52) knew that all three medicaments chloroquine, artesunate, and mefloquine were anti-malarials. 6% (3/52) knew that also doxycycline had anti-malarial activity. 5) 81% (42/52) were aware that the duration of the treatment can be dependent on the type of the parasite, and 6) 48% (25/52) knew three methods for preventing malaria (e.g. impregnated mosquito nets, fumigation/indoor spraying, and avoiding breeding sites).

Educational level and type of work were correlated to knowledge about malaria. When stratified for academic level, 20% (3/15) of unskilled workers, 67% (14/21) of employees with A-level (advanced training after secondary school), and 69% (11/16) of technical staff, respectively, identified the three main malaria parasites present in Venezuela. When stratified for medical or non-medical employees (inspectors/microscopists/visitors vs. fumigators/drivers/secretaries), 84% (21/25) of those working in medical activities, and 44% (12/27) of those working in non-medical activities, respectively, knew that artesunate is an anti-malarial drug.

### Treatment and adherence

Malaria treatment in the Venezuelan Amazon is exclusively in the hands of the malaria surveillance system. No malaria medicaments are sold in pharmacies and there are no other sources of anti-malarial drugs. Little is known about the impact of drugs crossing the border from neighbouring Colombia or Brazil. However, as the public health system of Venezuela provides cost-free treatment, in any event, it can be assumed that traffic of medicaments is rather from Venezuela to neighbouring countries than vice versa. In 2004, the Venezuelan Health Ministry established artemisinin-based combination therapy (ACT) as first-line treatment for *P. falciparum *(Artesunat^®^, Mefloquine^®^, and primaquine). For the treatment of *P. vivax*, chloroquine and primaquine is applied. ACT had a visible impact on transmission of *P. falciparum *(Figure 5). The impact on *P. vivax *was less pronounced (Figure 6).

The guidelines of the malaria control programme imply that treatment is taken under the supervision of health personnel. However, due to long distances and socio-cultural conditions, this is often impossible. Thus, little is known about adherence to malaria treatment and its impact on the development of resistance due to sub-therapeutic dosages by incomplete treatment regimens.

### Detection of cases

Malaria surveillance in Amazonas is based on the early diagnosis of cases through active and passive case detection. Passive detection is realized through 82 NPs, located in health posts, hospitals, consultancies and mission posts. Most of them are run by microscopists, who also administer treatment. All medical professionals, including private doctors, are obliged to send suspected malaria cases to the nearest NP. Active case detection is realized by 24 visitors. These health workers are sent to areas and communities with high incidence of malaria or where epidemics are anticipated. Besides active and passive case detection, 150 voluntary community health workers trained by PAMAFRO (see above) contribute to the detection of malaria cases.

**Figure 9:**
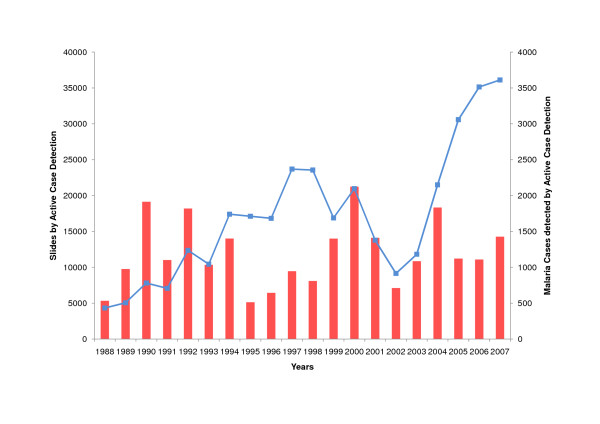
**Active Case Detection in Amazonas (1988 - 2007)**. Slides taken by active case detection (blue, left vertical axis), cases detected by active case detection (red, right vertical axis).

**Figure 10:**
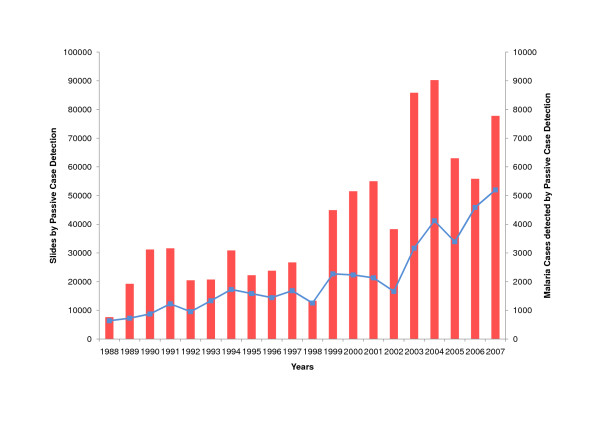
**Passive Case Detection in Amazonas (1988 - 2007)**. Slides taken by passive case detection (blue, left vertical axis), cases detected by passive case detection (red, right vertical axis).

The proportion of slides taken by active and passive case detection did not change significantly during twenty years: in 1988, 40% (4330/10736) of slides were taken by active case detection, and in 2007, 41% (36112/80969) of slides were taken by active case detection. Actively detected cases did not increase with the increase in the absolute number of actively taken slides (r = 0.24, p = 0.29) (Figure 9), However, the increase of passively taken slides correlated significantly with the number of passively detected cases (r = 0.87, p < 0.001) (Figure 10). In 2007, only 27 cases (0.3%) were registered as cases detected by voluntary community health workers trained by PAMAFRO.

### Quality assessment of malaria diagnosis

#### Microscopy

In 2007, 88 079 microscopic slides were examined in Amazonas (Figure 2). The malaria surveillance system stipulates a double quality check: All positive slides and 10% of negative slides are to be re-examined by microscopists in the Central Malaria Laboratory (CML) in Puerto Ayacucho. Then, these slides are sent to the National Malaria Reference Laboratory in Maracay, Venezuela, for a second re-examination. The concordance of slide reading between health posts and CML was reported to be 99%. However, the slides in the CML were re-examined in a non-blinded manner. A pilot study was carried out in order to read 1,000 slides in a blinded way. Results showed a lower concordance (Cohens Kappa Index = 0.74) and considerable differences between particular microscopists in the health posts due to different levels of refresher training.

#### Rapid Diagnostic Tests (RDTs)

In 2007, nearly 7,000 RDTs were given out by PAMAFRO to microscopists and health workers for use and evaluation. However, it was observed that many microscopists ceased microscopy and used RDTs only. This was in stark contrast to the low confidence of microscopists in RDTs, because it was known that the performance of RDTs is not optimal when the parasitaemia is low and storage conditions are inadequate (as is the case in most areas of Amazonas). In order to improve quality assessment and confidence in the adequate routine use of RDTs, a pilot study with the systematic evaluation of 500 RDTs was performed. Results showed that concordance with microscopy was good, but not optimal (Cohens Kappa Index = 0.72) (manuscript in preparation).

### Documentation system

The flow of information and the documentation of activities is mainly handled with paper and pencil by the local malaria teams. Many records are kept as in Arnoldo Gabaldón's times (see above). Digitalization has been introduced at some places for practical reasons, but a more systemic approach is required. Thus, many data of high quality, for example data for temporal and spatial transmission (Figures 5 and 6), are saved impeccably, but they are not easily accessible to modern research methods. In workshops it has been agreed that a great number of superfluous formats could be eliminated, and others have been transformed into a single format format. Training courses for staff members in data management and processing have been planned and implemented.

An important factor concerning the flow of information is the limited infrastructure. When transport is possible only via river or air, news from far away health posts depends on weather conditions and available logistics. Normally, written data and slides arrive within a week in the capital Puerto Ayacucho. As malaria cases in far away health posts are also reported via radio communication, another epidemiological data base is created using this route. This data base sometimes includes data slightly different from the malaria surveillance documentation, since the latter, for example, includes only double-checked cases.

### Entomology and vector control

Entomological activities comprised supervision of breeding sites including investigations for the evaluation of biological larvicides (*Bacillus sphaericus*). Breeding sites were mapped on paper formats. Night catches are carried out throughout the year, but are limited by logistic constraints. In 2007, 1,016 *Anopheles darlingi *(2006: 3252) and 574 *Anopheles braziliensis *(2006: 1622) were caught and identified. Roughly half of the mosquitos were captured indoors. Peaks of night catches coincided with peaks of rain during the months of September, October, and November.

Vector control activities comprised indoor residual spraying and fumigation. Sprayings and fumigations were carried out in endemic areas or where epidemics were anticipated. Anticipation of epidemics is calculated by inspectors through the analysis of local malaria epidemiology in the respective communities. In 2007, 5,288 houses were sprayed (2006: 3,350) and and the fumigation of 339,009 houses was performed (2006: 396 348). The insecticides used were fenothion powder (2007: 7,107 kg), fenothion emulsion (2007: 10,920 litres), deltamethrin (2007: 9,153.7 litres) for spraying of houses. For fumigation, malathion (2007: 18,078.32 litres), and fenothion (2007: 11,187 litres) were used.

The population of Amazonas is familiar with ITNs, but the coverage of effective bed net usage is rarely documented [14]. In 2007, 10,000 impregnated mosquito nets were distributed in the municipality of Atures, mainly in indigenous communities. The analysis of its impact will be described elsewhere.

## Discussion

Health care for malaria patients in Amazonas is guaranteed and mortality from malaria is low. However, malaria, as dengue fever, is a sensitive issue in Venezuela. The rise and fall in malaria cases serves as a topic for the politics of the day. In this context, it is important to show that malaria epidemiology is dependent on many factors and thorough analysis is needed to describe these trends and explain the reasons. A first glance at the malaria surveillance data of Amazonas shows a rising number of malaria cases during the last two decades and, even when the rise in population is considered, the Annual Parasite Index (API) is increasing. However, at the same time the number of notification points (NPs) increased as well, and the positivity rate of slides (SPR) maintained a steady state for the last two decades.

Moreover, subanalysis of species reveals that the proportion of *P. falciparum *cases actually decreased, whereas *P. vivax *stayed at the same level. Thus, host immunity, malaria surveillance and parasite transmission has kept a balance over the years and the existing risk of getting malaria has been accompanied by low mortality. In respect of *P. falciparum*, this equilibrium was altered positively by the introduction of ACT.

In 2003, the malaria surveillance system in the state of Sucre - the third federal state of Venezuela endemic for malaria, behind Amazonas and Bolivar - reported success reducing the API from 20,1 to 6,1. This was mainly the result of Roll Back Malaria interventions. Temporal and spatial transmission patterns had been analysed and mass treatment and integrated vector control measures, accompanied by health education, community participation and an overall strentgthening of the health services, had been carried out [32-35].

A key issue in public health is equity, which can be established through access to diagnosis and treatment. Ensuring that a high proportion of patients have access to effective treatment will be essential to malaria elimination programmes [36]. In Amazonas, the traditional malaria surveillance system is backed by the government's decision to provide free health care for all. It can be stated that - with the exception of the part of the Yanomami population living reclusively in remote areas of the *Alto Orinoco Casiquiare *Biosphere Reserve - free access to malaria diagnosis and treatment is provided for the entire population.

Apart from the biosphere reserve, the Venezuelan Amazon displays two zones of malaria transmission: the more densely populated northern part shows a seasonal pattern with peaks of malaria cases lagging three to four months behind rainfall peaks. This area is characterized by a savanna-like geography and easy access to health posts. Communities in the less densely populated southern part, live alongside rivers at the edge of the rainforest. Patients often have to reach a health post by foot or by boat. No seasonal pattern of malaria transmission could be detected here. This lack of seasonality was also noticed in an earlier epidemiological study in *Alto Orinoco *[17].

One important finding was that ethnic groups display different risks for malaria. This might be the consequence of differing socio-economic conditions, living habits and patterns of adherence to malaria drugs. For example, the *criollo *(mixed) population living in the urbanized area of Puerto Ayacucho showed a much lower risk for malaria than the indigenous Guahibo living in rural areas around the city. Future interventions should focus on these "hot spots" of transmission. As a consequence of this, a research project for the evaluation of blister packed treatment was implemented in 2008, which considers age and weight as well as the ethnic background of the patients.

The purpose of this RMA and provision of technical assistance was to provide baseline data for possible interventions as well as to define and facilitate reorganization, if it is necessary. In initial interviews with key players it was agreed that the focus of this project should be the evaluation of the surveillance data and the assessment of malaria diagnosis. Cross-sectional school parasitaemia and health-facility based surveys - as proposed in the rapid urban malaria appraisal (RUMA) methodology of African cities [37] - were not prioritized in this RMA, because the monitoring system in Amazonas functions adequately and the quality of the routine health statistics was found to be high. Thus, it could be assumed that all suspected cases were captured and confirmed microscopically.

It is appropriate to discuss and plan the final design of a RMA and technical assistance project in a horizontal manner with the actors involved. As conditions differ locally the methodology has to be adapted accordingly [38]. Fixed protocols appear advantageous at first glance, but some elements might turn out to be unfeasible under resource-constrained conditions. For example, mapping of breeding sites might be possible only under certain conditions [39-42]. Hence, in this RMA it was decided that the entomological and vector control unit would be subject to preliminary evaluation only, to be followed by a separate evaluation project.

Small steps - appearing unimpressive at first sight - were crucial for later success. For example, before carrying out the blinded double-check of field slides, microscopists had to be convinced that conflicting results from two readers are nothing to be ashamed of. Also, the digitalization of a hand-written documentation system, which had stood the test of time for half a century, had to be discussed in detail with the personnel involved and requires a long-term perspective. As possible reorganization of operational structures, such as effective quality assessment of microscopy and digitalization of the information system, would not take place before the end of an evaluation project, further monitoring is indicated.

The fight against malaria is highly dependent on the participation of communities. It has long been known that community health workers (CHW) are key figures in the interplay between the state's health system and communities [43]. In this context, Venezuela looks back to a long tradition of training CHWs [44]. Interestingly, by now, two different approaches were observed within the malaria surveillance system of Amazonas. On one hand, salaried state health workers were trained as in many other parts of the Venezuelan health system [45] and, on the other hand, non-paid CHWs were trained following concepts of voluntary participation (see PAMAFRO) creating ambiguities also seen in other parts of Venezuela [35].

A voluntary approach might be easily brokered to communities living in regions where the public health system has been commercialized or does not exist, as is the case in parts of Africa. However, it might be necessary to modify this non-governmental approach in countries opposing neoliberal reforms and showing the political will to establish professionalized health care, through which CHWs can make a living [46]. Probably, these conflicting approaches explain the observed suboptimal performance of voluntary CHWs in respect to slides taken. In order to stimulate voluntary participation it might be worth considering avoiding the overlap of activities between professional and voluntary CHWs. Slide taking was only one of various activities by voluntary CHWs and a complete analysis is underway.

Another clash of strategies was observed in respect to the distribution of RDTs to CHWs and microscopists. The use of RDTs saves lives and reduces overtreatment in the African scenario, where microscopy is scarce. However, when a network of trained microscopists exists, the distribution of RDTs might cause microscopy to be neglected as was seen in this RMA. In consequence, skills of trained microscopists and health workers could be lost rapidly. As low level parasitaemia is common in the Venezuelan Amazon and reaches the detection limit of both methods, microscopy and RDTs [18], the introduction of RDTs has to be carefully undertaken and monitored. In this scenario it may be preferable to improve microscopy rather than replace it.

In conclusion, the example of the Venezuelan Amazon shows that - unlike many African countries, where impoverishment and privatization have created different conditions for health interventions - the control of malaria is possible, at least to a certain degree, and in the case that health facilities are working satisfactorily. The significant decrease in slides taken in the years 2002 and 2003 (Figure 2) shows how sensitively health parameters react to socio-political circumstances: these years were charaterized as the years of a failed coup d'etat and of the economic paralysis of the country. Other public health parameters in Venezuela, for example child mortality, also experienced a clear negative impact in this period [47].

This RMA of Amazonas showed that, despite various shortcomings and logistic problems because of limited infrastructure, the malaria surveillance system in the Venezuelan Amazon provides a solid foundation for further interventions. The estimation whether eradication or elimination of malaria will be possible in the Venezuelan Amazon or may, rather, be a distant prospect, was outside the scope of this RMA.

## Abbreviations

ACT: Artemisinin-based Combination Therapy; API: Annual Parasite Index; CHW: Community Health Worker; CML: Central Malaria Laboratory in Puerto Ayacucho; ICD: International Classification of Diseases and Related Health Problems; ITN: Insecticide Treated Bed Nets; NP: Notification Point; PAMAFRO: *Plan Andino de Malaria en Areas Fronterizas *(Andean Malaria Plan in Frontier Areas); RDT: Rapid Diagnostic Test; RMA: Rapid Malaria Appraisal; RUMA: Rapid Urban Malaria Appraisal; SPR: Slide Positivity Rate.

## Competing interests

The authors declare that they have no competing interests.

## Authors' contributions

WGM conceived the study, coordinated and conducted the field work, analysed and interpreted data, and drafted and revised the manuscript. AMG participated in the conception of the work and coordinated the field work. SVM participated in the conception of the work, interpreted the data, and revised it critically at all stages. JG was the key local contact person; he supervised the field activities. JAC was the administrator of the documentation system and participated in the cleaning of data sets. BGM contributed to the statistical analysis and revised the paper. MM facilitated the overall collaboration and participated in the conception of the work. All authors read and approved the final manuscript.

## Supplementary Material

Additional file 1 **Questionnaire**. The questions about malaria knowledge were part of a larger questionnaire evaluating operational and organisational aspects and problems of the malaria surveillance system in Amazonas. They were translated from Spanish and are not in the original design. Click here for file
