# Supplementary material for: A rapid malaria appraisal in the Venezuelan Amazon
Source: Malar J. 2009 Dec 11;8:291. doi: 10.1186/1475-2875-8-291 (PMC2799431; doi:10.1186/1475-2875-8-291)
Supplement: Additional file 1 — Questionnaire. The questions about malaria knowledge were part of a larger questionnaire evaluating operational and organisational aspects and problems of the malaria surveillance system in Amazonas. They were translated from Spanish and are not in the original design. [file 1475-2875-8-291-S1.PDF]

Questionnaire (translation from spanish, not in original design):

The following are the questions which were analysed for the Rapid Malaria Appraisal (questions 1-6, 25-30). They were part of a larger questionnaire evaluating operational and organisational aspects and problems of the malaria surveillance system of Amazonas.

With the aim to gather ideas for the improvement of this institution, we would like to ask for your help to answer the following questions.

1) Age______ *Sex______*

2) Academic level:

a) Primary school: complete incomplete
b) High school: complete incomplete
c) Median technician
d) Superior technician
e) Bachelor degree or equivalent:
f) Others:_____________________________________

3) Date of beginning of work:

4) Labor condition:

a) Short-term contract
b) Permanent

5) In case of a permanent position. How did you get the job?

a) Contest
b) Assignation

*6) Which kind of work are you carrying out at the moment?*________________________________

---

7 – 24 (not translated)

Questions about malaria. Mark the answer which you consider correct (you can mark more than one answer).

25) How is malaria transmitted?

a) through contaminated water
b) through the air
c) through mosquitos
d) through god

26) Which malaria parasites exist in Venezuela?

a) Plasmodium falciparum
b) Plasmodium vivax
c) Plasmodium ovale
d) Plasmodium malariae

27) Which of these parasites is the most dangerous ?

a) Plasmodium falciparum
b) Plasmodium vivax
c) Plasmodium ovale
d) Plasmodium malariae

28) Which of the following drugs are antimalarial drugs (which can eliminate the parasites)?

a) Chloroquine
b) Aspirine
c) Doxycycline
d) Artesunate
e) Mefloquine

29) How many days has an antimalarial treatment to be administrated?

a) one day
b) two days
c) three days
d) 14 days
e) It dependents on the species of parasite which is diagnosed

30) How can malaria be prevented?

a) cooking water
b) eliminating water around the house
c) using mosquito nets
d) fumigating
